# Evaluation of the better operative outcome software tool to predict cataract surgical outcome in the early postoperative follow-up

**DOI:** 10.1186/s12886-023-03058-1

**Published:** 2023-07-13

**Authors:** M. Lecumberri, C L. Moser, J. Loscos-Arenas

**Affiliations:** 1 Complex Hospitalari Universitari Moisès Broggi, Barcelona, Spain; 2 Eyes of the world Foundation, Barcelona, Spain; 3 Hospital Germans Trias i Pujol, Universitat Autónoma de Barcelona, Barcelona, Spain; 4 Proyectovision NGO, Barcelona, Spain

**Keywords:** Cataract outcome, Monitoring, e-Health, Avoidable blindness, Early assessment

## Abstract

**Background:**

Cataracts are the world’s leading cause of avoidable blindness. In low-income countries, there are high rates of poor follow-up, which makes it very difficult to monitor surgical outcomes. To address this issue, the Better Operative Outcome Software Tool (BOOST Cataract app) predicts outcome on the first postoperative day and provides specific advice to improve outcomes. The aim of the study is to evaluate the ability of the BOOST Cataract app to categorise surgical outcomes and to analyse the possible factors that contribute to its performance. This was a prospective observational study performed at the General Hospital of Hospitalet of Llobregat.

**Results:**

A total of 126 cataracts were included. Patients had a mean [SD] age of 75.8 [12.19] years, and 52% were females. Manual small-incision cataract surgery was involved in 57% and phacoemulsification in 43%. Thirty-eight percent of eyes presented significant corneal oedema on day 1. The BOOST Cataract app succeeded in categorising the final outcome in 65.6% of the eyes and in 93,4% of the eyes with good outcome.The agreement between the BOOST and UDVA outcomes was 0.353 (*p*< .000*).* The level of agreement improved to 0.619 (*p*< .000) in eyes with clear corneas. Success obtained by BOOST for both types of surgery was not statistically different. Eyes that obtained a good outcome on day one after surgery and eyes with clear cornea had 37 times higher odds (95% CI 6.66, 212.83) and 12 times higher odds (95% CI 3.13, 47.66) of being correctly categorised by the BOOST Cataract app than eyes that obtained a suboptimal (moderate and poor) outcome and eyes with corneal oedema on day 1.

**Conclusions:**

The BOOST Cataract app is an e-Health tool designed to address issues of measuring quality in low- and middle-income settings. Although its reliability is limited to eyes that obtain a good outcome and with clear corneas on day 1, the use of the tool on a regular basis facilitates monitoring and reporting outcomes when clinical data collection is challenging due to low postoperative follow-up rates.

## Background

Cataracts remains the world’s leading cause of avoidable blindness, affecting 15.2 million people, 90% of whom live in low-income countries [1]. Vision loss negatively impacts individuals, households and communities, increasing poverty and reducing quality of life [2]. Quality cataract surgery is a highly cost-effective procedure for reducing the impact of blindness and visual impairment.

There are multiple reports of poor outcomes after cataract surgery in low-income and middle-income countries (LMICs). A systematic review undertaken in October 2021 showed the proportions of participants with postoperative presenting visual acuity (VA) ≥ 0.32 (20/60) at below 70% in LMICS studies, with a range of 29.9–80.5% [3].

In many developing countries, postoperative follow-up is only 20–30% of patients. These low follow-up rates make it very difficult to monitor surgical outcomes [4]. Different approaches have been proposed for estimating the final visual outcome after surgery without requiring follow-up based on measuring postoperative visual acuity or refraction on day 1 after surgery without waiting for complete postoperative treatment of the eye [5, 6].

In 2018, a global consortium of leading eye health organisations (the Fred Hollows Foundation, Sightsavers, The International Agency for the Prevention of Blindness, ORBIS International, Aravind eye Hospitals and the International Council of Ophthalmology) launched an application to address the issues of surgical quality measurement and surgical data recording and to help improve cataract surgical outcomes known as the *Cataract Better Operative Outcomes Software Tool* (BOOST Cataract app). The BOOST Cataract app is a free application and can be used for recording and analysing surgical outcomes the day after surgery. It has been designed to monitor cataract surgery performed by phacoemulsification, manual small incision cataract surgery or corneal extracapsular cataract extraction. Users can compare their performance against other users around the world**.** There are two phases in the assessment process. In Phase I, preoperative best corrected distance visual acuity (BCDVA) and postoperative uncorrected distance visual acuity (UDVA) are registered on days 1 to 3 after surgery, and visual outcome is categorised according to the World Health Organisation (WHO) benchmark threshold of reference for effectiveness as a good, moderate, or poor outcome (Table 1), [7] thus estimating the final outcome. The tool assumes that the patient's visual acuity on days 1 to 3 after surgery is a valid indicator of the final outcome, thereby overcoming the issue of lack of follow-up that prevents a good assessment of the surgical outcomes. UDVA is preferred over BCDVA because it is closer to presenting visual acuity (PVA) where access barriers to optical correction exist [8].

**Table 1:** World Health Organization criteria

**Cataract surgical outcome according to postoperative visual acuity (7)**		
**Outcome**	**LogMar Visual acuity**	**Snellen Visual acuity**
Good	≤ 0, 5 LogMar	≥ 6/18
Moderate	> 0, 5 ≤ 1 LogMar	< 6/18 ≥ 6/60
Poor	> 1 LogMar	< 6/60
**Definition of distance visual impairment and blindness (ICD 11)**		
Mild visual impairment		< 6/12
Moderate visual impairment		< 6/18
Severe visual impairment		< 6/60
Blindness		< 3/60

Phase II consists of analysing the possible causes of poor outcomes. For this, UDVA and best corrected distance visual acuity (BCDVA) of 20 patients with a poor outcome 6 weeks after surgery are registered, and the possible aetiology is assessed: selection, complication, and refractive error. Based on this, the tool assumes the main cause of poor outcome.

Although the BOOST Cataract app is being used to monitor surgical outcomes in different settings [9], to our knowledge, the ability of the app to categorise surgical outcomes has not yet been analysed.

### Study aim

The aim of the study is to evaluate the ability of the BOOST Cataract app to categorise surgical outcomes and to analyse the possible factors that contribute to its performance.

### Implementation

#### Participants and setting

The study included 126 cataract surgeries performed at the General Hospital of Hospitalet of Llobregat (Barcelona) from June 2020 to December 2021.

Cataracts were classified according to the LOCS III classification system and recruited by convenience sample (NC5 and NC6). NC6 patients were enrolled in small manual incision cataract surgery (MSICS), and NC5 patients were allocated for phacoemulsification. Exclusion criteria were absence of pupillary light reflex and retinal pathology demonstrated by fundoscopy (NC5 cataracts) or ultrasound (NC6 cataracts).

Informed consent was provided by all patients undergoing surgery. The study adhered to the guidelines of the Declaration of Helsinki. The protocol was approved by the institutional review board of Bellvitge University Hospital, Barcelona.

#### Preoperative examinations

All patients underwent a complete ophthalmological examination that included BCDVA, slit-lamp examination, pupillary reflexes, Goldmann applanation tonometry, and fundus examination when lens opacity allowed it. Ocular B-mode ultrasound (Echoscan US-4000 Nidek, Japan) was performed to rule out any pathology in the retina and vitreous in NC6 cataracts in which fundus examination could not be performed. The power of the intraocular lens (IOL) implanted was calculated using the IOL-Master 500 (Carl Zeiss Meditec, Jena, Germany) when lens opacity allowed it and by keratometry and ocular mode-A ultrasound (Echoscan US-4000 Nidek, Japan) in NC6 cataracts.

#### Visual acuity assessment

Visual acuity (VA) was assessed using Tumbling E charts for distance vision using the patient's best optical correction. After correctly identifying the direction of more than half of the optotypes in the upper line, the patient was moved to the next line and successively to lower lines. The lowest line in which more than half of the optotypes were read was recorded as the patient's VA. The patient was moved to half the distance when not able to identify the direction of any line. If the patient was not able to identify the optotypes at that distance, the assessor’s fingers were placed at distances of 1 m and 50 cm from the patient. If the patient was unable to see the fingers, a light source was placed to document light perception.

VA was measured on a Snellen scale and then converted to a LogMAR scale. The VA of light perception was considered 0.0020 (LogMAR 2.70) [10].

#### Surgery and postoperative treatment

All surgeries were performed by two ophthalmologists with extensive experience in MSICS and phacoemulsification procedures (R.A.B. and M.L.L). MSICSs were performed by making a U-shaped frown scleral incision to minimise surgically induced astigmatism [11]. A Tecnis ZCB00 monofocal 1-Piece foldable IOL (Johnson & Johnson Vision, Inc., USA) was implanted in patients who underwent phacoemulsification, and a rigid PMMA EZE.60 IOL (Baush & Lomb, Inc., USA) was implanted in patients who underwent MSICS. All patients received perioperative prophylaxis with intracameral 0.1 mL cefazolin (2.5 mg/mL) or 0.1 mL vancomycin (0.1 mg/mL) if allergic to penicillin at the end of surgery [12].

All patients followed the same postoperative treatment protocol at the hospital, consisting of 1 drop of Vigamox® (Moxifloxacin 5 mg/mL; Lab Alcon Cusi, Spain) every 8 h during the first week, tapering doses of Maxidex® (Dexamethasone 1 mg/mL, Lab Alcon Cusi, Spain) for 3 weeks (1 drop every 8 h during the first week, 1 drop every 12 h during the second week, and 1 drop every 24 h during the third week), and 1 drop of Diclofenaco Lepori monodosis® (Diclofenac sodium 1 mg/mL; Lab Angelini, Spain) every 8 h for 6 weeks.

#### Assessment the day after surgery and after 6 weeks

Patients were examined the day after surgery, and VA was measured in the operated eye by an independent observer (BOOST VA). Slit-lamp examination was used to determine corneal oedema. Significant corneal oedema was defined as more than 10 Descemet folds or stromal corneal oedema affecting the visual axis.

VA was measured again 6 weeks after surgery without correction (UDVA) by an optometrist masked to the VA measured on day one. Visual acuities were classified according to the WHO criteria (Table 1) on day 1 after surgery (BOOST outcome) and 6 weeks after without optical correction (UDVA outcome).

#### Statistical analysis

##### Sample size calculation

Differences in percentages of BOOST outcome and UDVA outcome were considered for the sample size calculation since this comparison was the main objective of the study. Accepting a confidence level and power of 95%, a minimum of 116 subjects were required to detect a difference equal to or greater than 20%. A loss to follow-up rate of 10% was estimated.

Descriptive analysis was performed showing the absolute and relative frequencies for qualitative variables, means and standard deviations for normally distributed continuous variables and medians and interquartile ranges for nonnormal continuous variables. Categorical data were analysed using the chi-squared test. Likelihood ratios were used when at least 20% of the cells had expected values lower than 5. The Kolmogorov‒Smirnov test was used to test the normal distribution of quantitative variables.

The Mann‒Whitney U test was used to compare medians between two groups, and the Kruskal‒Wallis test was used for three or more groups.

Agreement between BOOST outcome and UDVA outcome was assessed using Cohen’s kappa agreement test. Agreement was calculated separately for outcome obtained at day 1 and for eyes with clear cornea and corneal oedema on day 1. A statistically significant Cohen’s kappa agreement estimate indicated the concordance of both methods.

BOOST outcome and UDVA outcome were transformed into numerical variables following the following criteria: good = 1, moderate = 2, or poor = 3. The success obtained by BOOST was calculated by creating a new variable resulting from the subtraction of the value obtained at 6 weeks and the value obtained by BOOST: when the result of the subtraction was zero, it was considered a success, and when it was different from zero, it was considered an error. The proportion of success obtained by BOOST and separately for outcome at day 1, type of surgery and corneal status on day 1 were calculated.

To identify factors associated with success obtained by BOOST, a binomial logistic regression analysis was performed. The dependent variable was the success obtained by BOOST (with 2 categories: success and error), and the predictor variables were the type of surgery, presence of oedema in the early postoperative period, postoperative astigmatism, spherical equivalent and BOOST outcome (good and suboptimal).

The significance level was set at 0.01 in all cases. The estimations of the parameters are shown along with their 95% confidence intervals. All analyses were performed in SPSS v.24 (IBM Corp., Armonk, NY, USA).

## Results

The study included 126 patients who underwent cataract surgery. The mean [SD] age of the patients was 75.8 [12.19] years, and there were 66 (52.4%) female patients. Fifty-five eyes (43.6%) were left eyes. MSICS was performed in 57% (*n* = 72). A total of 59 contact biometries were performed (89%, MSICS group). Both groups were equal regarding age and gender. The preoperative VA was 1.4 LogMar (ST = 0.98). Patients in the MSICS group showed a worse preoperative VA than patients in the phacoemulsification group. Four patients did not complete the study and hence were excluded (Table 2)*.*

**Table 2:** Characteristics of the sample

**Preoperative data**					
		**Total** ***N* = 126**	**Phacoemulsification** ***N* = 54**	**MSICS** ***N* = 72**	***p* value**
**Age**					
Mean ± SD		75.1 ± 12.1	75.1 ± 8.8	76.4 ± 14.3	0.558^a^
Median		77	75	79.5	
IRQ		18	14	21.5	
**Gender**					
Male	n	60	31	29	0.057^b^
	%	47.6	57.4	40.3	
Female	n	66	*n* = 23	43	
	%	52.4	42.6	59.7	
**Preop VA LogMar**					0.0005^a^
Mean ± SD		1.40 ± 0.98	0.58 ± 0.48	2.01 ± 0.81	
Median		1	0.4	2.4	
IRQ		2.23	0.2	1.5	
**Day 1 postoperative *n* = 126**					
		**Total**	**Phacoemulsification**	**MSICS**	***p* value**
**BOOST VA LogMar**		***N* = 126**	***N* = 54**	***N* = 72**	
Mean ± SD		0.76 ± 0.66	0.49 ± 0.46	0.94 ± 0.69	0.002^a^
Median		0.5	0.4	0.7	
IRQ		0.7	0.5	0.9	
**Cornea status**					0.186^b^
Clear cornea	n	78	37	41	
	%	61.9	68.5	43.1	
Corneal oedema	n	48	17	31	
	%	38.1	31.5	56.9	
**Boost outcome**					0.0005^a^
Good	n	67	38	29	
	%	53.2	70.4	40.3	
Moderate	n	35	12	23	
	%	27.8	22.2	31.9	
Poor	n	24	4	20	
	%	19	7.4	27.8	
**6 weeks postoperative *n* = 122**					
		**Total** ***N* = 122**	**Phacoemulsification** ***N* = 53**	**MSICS** ***N* = 69**	***p* value**
**UDVA LogMar**					0.0005 ^a^
Mean ± SD		0.46 ± 0.53	0.17 ± 0.15	0.67 ± 0.61	
Median		0.3	0.15	0.5	
IRQ		0.35	0.26	0.7	
**Postoperative Astigmatism**					0.41^a^
** Mean ± SD**		-1.7 ± 1.3	-1.8 ± 1.4	1.8 ± 1.2	
** Median**		-1.5	-1.5	-1.25	
** IRQ**		2	1.75	2	
**Spherical equivalent**					
** Mean ± SD**		-0.49 ± 1.04	-0.3 ± 0.7	-0.6 ± 1.2	0.06^a^
** Median**		-0.5	-0, 5	-0.58	
** IRQ**		1	0.75	1.5	
**UDVA outcome**					0.0005
Good	n	93	53	40	
	%	73.8	98.1	55.6	
Moderate	n	16	0	16	
	%	12.7	0	22.2	
Poor	n	13	0	13	
	%	10.3	0	18.1	

*SD* Standard deviation, *%* Percentage, *IRQ* Interquartile range, *MSICS* Manual small-incision cataract surgery, *BOOSTVA* Visual acuity obtained ON day 1 postop, *UDVA* Uncorrected distance visual acuity after 6 weeks postop. ^a^ Mann Whitney U test. ^b^Chi-Squared test

Overall, surgical outcomes on day 1 were different than final outcomes (Table 3)*.* Surgical outcomes, both BOOST and UDVA, were superior for patients in the phacoemulsification group. The mean (SD) BOOST VA was 0.75 (0.66): 0.95 (0.71) in the MSICS group and 0.49 (0.47) in the phacoemulsification group. UDVA was 0.49 (0.57), 0.73 (0.66) for MSICSs and 0.17 (0.15) for phacoemulsification (*ƿ* < 0.0005).

**Table 3:** Success of BOOST categorising final outcome

	**%**	**95% CI**	***p* -value**
**Total**	65.6	(57.17; 74.02)	
**BOOST Outcome**			< 0.001^a^
Good	93.9	(99.85;87.94)	
Suboptimal (Moderate and Poor)	44,6	(67.56;21.63)	
**Type of surgery**			0.251^b^
Phacoemulsification	71.7	(83.82; 59.57)	
MSICS	60.9	(72.41; 49.38)	
**Status of the cornea on day 1**			0.0005^b^
Clear cornea	84.6	(76.84; 92.71)	
Corneal oedema	30.4	(17.1; 43.69)	

^a^Cohen´s Kappa ^b^Chi- Squared test

The BOOST Cataract app succeeded in categorising the final outcome in 65.6% of the eyes. It was found a statistically significant difference in success depending on BOOST outcome and status of the cornea at day 1. Success obtained by BOOST for both types of surgery were not statistically different (Table 3)*.*

The agreement between the BOOST and UDVA outcomes was 0.353 (*ƿ* < 0.0005*).* The level of agreement improved to 0.619 (*ƿ* < 0.0005) in eyes with clear corneas. Agreement was not significant in eyes that presented corneal oedema on day 1 (Table 4).

**Table 4:** Agreement between BOOST and UDVA outcome

	**Kappa**^a^	**SE**	**95% CI**	***p* -value**	**n**
**Total sample**	0.353	0.069	(0.48;0.21)	< 0.0005	122
**Corneal oedema on day 1**	0.082	0.073	(0.22; -0.06)	0.247	46
**Clear cornea on day 1**	0.619	0.107	(0.82;0.40)	< 0.0005	76

^a^Cohens´s Kappa coefficient. *SE* Standard error. *CI* Confidence interval

The results of bivariate logistic regression analysis showed that eyes that obtained a good outcome on day one after surgery and eyes with clear cornea had 37 times higher odds ( 95% CI 6.66, 212.83) and 12 times higher odds (95% CI 3.13, 47.66) of being correctly categorised by the BOOST Cataract app than eyes with suboptimal (moderate and poor) outcome obtained by BOOST Cataract app or eyes with corneal oedema on day 1. The odds of success of BOOST categorising UDVA outcome were not statistically significant for the type of surgery (phacoemulsification vs. MSICS) (Table 5).

**Table 5:** Factors associated with success of BOOST categorising UDVA outcome

	**Reference**	**OR**	**95% CI**	***P* value**
**Good BOOST outcome**	Suboptimal	37.65	6.66—212.83	0.0005
**Clear Cornea**	Corneal edema	12.22	3.13—47.66	0.0005
**Phacoemulsification**	MSICS	0.26	0.05—1.34	0.108
**Spheric Equivalent**		1.81	1.01—3.24	0.04
**Postoperative Astigmatism**		1.07	0.66 -1.72	0.773

R^2^ Nagelkerke = 0.68. *OR* Odds Ratio, *CI* Confidence interval

## Discussion

The BOOST Cataract app was created to monitor the surgical outcome of cataract surgery when there is no good patient follow-up. It is based on the PRECOG study, which demonstrated a correlation between visual acuity at early postoperative follow-up and at 40 or more days after surgery [6].

Our study shows that the agreement between the outcome obtained by BOOST and the real final UDVA outcome was only 35%, and success of BOOST was 65%. Agreement improved to 61%, and success increased to 86,4% in eyes with clear corneas on day one.

Corneal oedema is a sign of inflammation caused by endothelial dysfunction after surgery and is related to mature cataracts. It drastically, but temporarily, decreases vision, as it generally resolves without sequelae. Patients with a poor initial outcome due to corneal oedema will be categorised into the poor outcome group, although their VA will improve during the first month towards a good outcome. The tool underestimates the final outcome because it does not consider significant corneal oedema in the immediate postoperative period.

Similarly, the BOOST Cataract app was more reliable in categorising good outcomes than suboptimal (moderate and poor) outcomes: 93.9% of eyes with good BOOST outcomes preserved a good UDVA outcome. Patients in this group were relatively homogeneous: healthy eyes, minimal postoperative inflammation, and no complications. While only 44.6% of eyes with Suboptimal (moderate and poor) BOOST outcomes remained in the suboptimal UDVA outcome group.

BOOST VA and BOOST outcome were superior in the phacoemulsification group, but corneal oedema was not significantly different in either type of surgery (phacoemulsification and MSICS). For this reason, the error made by BOOST was not significantly different when considering both types of surgery separately.

The BOOST Cataract app was designed to be used for both phacoemulsification and the MSICS technique. Although MSICS has become the surgery of choice in LMICs, phacoemulsification is still used in those settings [13, 14].

This result validates the use of the BOOST Cataract app to monitor surgical outcome early in the postoperative stage for both types of surgery in settings with poor postoperative follow-up.

The user´s manual of the BOOST Cataract app specifies that postoperative VA can be registered from day 1 to 3 after surgery. We decided to measure postoperative VA only on day 1. This is because it more closely reflects the situation of low-income countries where most of the patients are admitted on the day of surgery and discharged the following day after the first postoperative visit, especially in outreach cataract campaigns. In settings with high barriers to access, vision on day 1 will be available, whereas vision on day 3 will be just as inaccessible as vision at 6 weeks [15, 16].

Taking into account that the ideal time to assess the surgical outcome is 6 to 8 weeks after surgery, the more time elapses from surgery to the final VA measurement, the closer the VA will be to the final result [17].

The creators of the BOOST Cataract app are aware of this issue and have proposed looser limits compared to those proposed by the WHO, thus allowing up to 35% of cases with moderate outcomes (instead of < 15% as recommended by the WHO) and consider a good outcome acceptable in 60% of patients (instead of the 80% recommended by the WHO) [18] (Table 6).

**Table 6:** Standards for postoperative visual acuity according to WHO and BOOST [13]

	**WHO standards** **(6 weeks after surgery)**	**BOOST standards** **(1–3 days after surgery)**
Good	> 80%	> 60%
Suboptimal		
Moderate	< 15%	< 35%
Poor	< 5%	< 5%

Postoperative astigmatism can play a role in UDVA; in our sample, it was not relevant. MSICS has often been discredited for inducing high astigmatism, but by adopting appropriate wound construction techniques, surgically induced astigmatism can be effectively controlled [11].

The final spherical equivalent was also irrelevant in our study. However, it can be an issue in achieving a good UDVA outcome in settings where the use of biometry prior to cataract surgery or an appropriately powered IOL is not available [19].

The main limitation of the study is that it was performed among individuals of a population in Barcelona. The objective of the study was to evaluate the ability of the BOOST Cataract app to categorise final outcomes, and the results should not be affected by the setting.

We have included NC6 cataracts to resemble the targeted population. Patients who underwent phacoemulsification had less advanced cataracts and better preoperative VAs than those who underwent MSICS since MSICS is used only for some NC6 cataracts in our practice. This is not the case in low-income countries, where it is common to operate on all cataracts using MSICS.

In our study, 100% of eyes with poor UDVA outcomes belonged to the MSICS group. Fifty-five percent (n = 11) had a poor prognosis before cataract surgery: 7 presented severe amblyopia, 4 presented advanced atrophic macular degeneration not detected prior to surgery, and 1 eye was subjected to cataract surgery to control pain for end-stage glaucoma due to pupillary blockage.

We did not exclude patients with amblyopia. The main objective of the study was to evaluate how well the BOOST Cataract app categorises final surgery outcome, especially in the MSICS procedure. In our environment, it is somewhat common to find patients with high amblyopic eyes and NC6 cataract consulting for other cataract-related symptoms such as glare rather than low visual acuity. Although the final VA outcome was poor, the patients’ symptoms improved after surgery [20, 21].

Capsule ruptures occurred in 6 eyes (100% MSICS group). Four of them had a clear cornea on day one after surgery and were categorised both by BOOST and after 6 weeks as having a good outcome (data not shown).

One hundred percent of late postoperative complications (Irvine-Gass syndrome, anterior ischaemic optic neuritis and central retinal vein occlusion) occurred in patients from the MSICS group, although there were no surgical complications in those patients. This may be because patients with NC6 cataracts in our practice more frequently have other ophthalmic or systemic pathologies compared to patients in low-income countries, where it is more common to find eyes with NC6 cataracts without other associated pathologies.

The BOOST Cataract app categorises outcome according to the WHO benchmark threshold of reference set more than 20 years ago. Good outcome is defined as a presenting visual acuity of 6/18 or better. In LMICs, since the shift from extracapsular cataract extraction to manual small-incision cataract surgery and the extended use of IOLs, it is more likely to achieve a good outcome [22].

Although reliability of BOOST Cataract app is fairly good, only for categorising good outcome, the mobile surgical outcome application facilitates the monitoring process in LMCIs and may play a roll in reporting outcomes where data collection is challenging due to low postoperative follow-up rates.

It remains to be seen how the findings obtained in this study might be affected when applied to the intended population, where even obtaining a reliable VA after surgery can be challenging due to the low resources.

## Conclusions

Reliability of the BOOST Cataract app was only fairly good in eyes with clear cornea on the early postoperative follow-up and in patients who presented a good outcome on day 1. It would be desirable to consider the presence of corneal oedema to improve reliability.

The BOOST Cataract app is an e-Health tool designed to address issues of measuring quality in low- and middle-income settings. Despite the tendency to underestimate surgical outcome, the use of the tool on a regular basis facilitates monitoring and may be useful to detect drops in surgical outcome, especially in outreach cataract campaigns that may need further posterior evaluation.

### Availability of requirements

The data have not been placed in any online data storage. The datasets used and/or analyzed during the current study are available from the corresponding author on reasonable request.

Project name: Better operative outcome software tools.

Project home page: https://boostcataract.org/home.html.

Operating system(s): Windows, Android.

Programming language: Not available.

License: Not available.

No restrictions to use by non-academics: Free access. Registration needed.

## Data Availability

The data have not been placed in any online data storage. The datasets used and/or analyzed during the current study are available from the corresponding author on reasonable request.

## Abbreviations

LMICS: Low-income and middle-income countries
WHO: World Health Organization
UDVA: Uncorrected distance visual acuity
BCDVA: Best corrected distance visual acuity
PVA: Presenting visual acuity
IOL: Intraocular lens
VA: Visual acuity
BOOST: Better Operative Software Outcomes Tool
MSICS: Manual small incision cataract surgery
